# GNA13 expression promotes drug resistance and tumor-initiating phenotypes in squamous cell cancers

**DOI:** 10.1038/s41388-017-0038-6

**Published:** 2017-12-19

**Authors:** Suhail Ahmed Kabeer Rasheed, Hui Sun Leong, Manikandan Lakshmanan, Anandhkumar Raju, Dhivya Dadlani, Fui-Teen Chong, Nicholas B Shannon, Ravisankar Rajarethinam, Thakshayeni Skanthakumar, Ern Yu Tan, Jacqueline Siok Gek Hwang, Kok Hing Lim, Daniel Shao-Weng Tan, Paolo Ceppi, Mei Wang, Vinay Tergaonkar, Patrick J. Casey, N. Gopalakrishna Iyer

**Affiliations:** 1 Programme in Cancer and Stem Cell Biology, Duke-NUS Medical School, Singapore, Singapore; 2 Cancer Therapeutics Research Laboratory, National Cancer Centre, Singapore, Singapore; 3 Mouse Models for Human Cancer Unit, Institute of Molecular and Cell Biology, Singapore, Singapore; 4 Department of Surgical Oncology, National Cancer Centre, Singapore, Singapore; 5 Advanced Molecular Pathology Laboratory, Singapore, Singapore; 6 Department of General Surgery, Tan Tock Seng Hospital, Singapore, Singapore; 7 Department of Pathology, Singapore General Hospital, Singapore, Singapore; 8 IZKF Junior Research Group, Friedrich-Alexander-Universitaet Erlangen-Nuernberg, Erlangen, Germany; 9 Department of Pharmacology and Cancer Biology, Duke University Medical Center, Durham, USA

## Abstract

Treatment failure in solid tumors occurs due to the survival of specific subpopulations of cells that possess tumor-initiating (TIC) phenotypes. Studies have implicated G protein-coupled-receptors (GPCRs) in cancer progression and the acquisition of TIC phenotypes. Many of the implicated GPCRs signal through the G protein GNA13. In this study, we demonstrate that GNA13 is upregulated in many solid tumors and impacts survival and metastases in patients. GNA13 levels modulate drug resistance and TIC-like phenotypes in patient-derived head and neck squamous cell carcinoma (HNSCC) cells in vitro and in vivo. Blockade of GNA13 expression, or of select downstream pathways, using small-molecule inhibitors abrogates GNA13-induced TIC phenotypes, rendering cells vulnerable to standard-of-care cytotoxic therapies. Taken together, these data indicate that GNA13 expression is a potential prognostic biomarker for tumor progression, and that interfering with GNA13-induced signaling provides a novel strategy to block TICs and drug resistance in HNSCCs.

## Introduction

Treatment failure (primary or secondary) is a significant cause of death in solid tumors. These failures manifest as resistance to “standard-of-care” treatment modalities or to the development of distant metastasis. In both scenarios, options are limited except in infrequent instances where there is a clear, druggable oncogenic driver as with the case in EGFR-driven lung adenocarcinoma or HER2-dependent breast cancers. Current evidence suggests that the ability of solid tumors to evade cytotoxic therapies (such as radio- and chemotherapy) is a direct function of intra-tumor heterogeneity [1]; tumor recurrence, resistance, and metastasis can be attributed to small, aggressive sub-populations of cancer cells that survive the onslaught of these modalities and eventually overwhelm the patient [2]. Various traits have been ascribed to these subpopulations, and there is significant debate as to whether the data can be generalized across all solid malignancies. Notably, these subpopulations have the ability initiate and recapitulate the entire tumor, and possess many of the attributes of stem cells, leading to their designation as tumor-initiating cells (TICs) [3]. In addition, some of these cells demonstrate a phenotype of having undergone epithelial-to-mesenchymal transition (EMT), with data suggesting a great degree of overlap between TICs and EMT phenotypes [4].

The identification of the TIC subpopulation of cancer cells have been aided by the use of surface markers, including CD44 in breast and head and neck, CD133 in colorectal and CD166 in lung cancers, respectively, and the activity of enzymes such as aldehyde dehydrogenase (ALDH1) [5–7]. Subpopulations identified using these markers have increased potential for tumor-initiation, distant metastases, and resistance to multiple cytotoxic drugs and radiation therapy [8]. Hence, there is significant interest in targeting these aggressive sub-populations through the inhibition of signaling pathways that drive the TIC phenotype [9]. To date, these efforts have focused on pathways such as transforming growth factor β, WNT-βCatenin, Notch, Hedgehog, PDGFR, and IL6, and have yielded some promising results [7]. What has emerged from these experiments is that EMT/TIC-phenotypes are critical cancer traits that can be targeted, but the pathways that control these phenotypes vary between tumors [1, 7]. Therefore, understanding the different mechanisms that support the growth of TICs specific to each tumor could identify an individualized “Achilles heels” that can be targeted to improve therapeutic outcomes for that tumor type.

G protein coupled receptors (GPCRs) are a large family of cell surface receptors, many of which have been implicated in cancers [10]. GPCRs such as CXCR4, LPAR, PAR1, LGR5, and S1PR are up-regulated in many advanced cancers and induce invasion and metastasis [11], while CXCR4 [12], CXCR1/2 [13] and LGR5 [14] have been linked to TIC-like phenotypes. Interestingly, most of these GPCRs signal at least in part through G12 proteins [15], a subfamily of G proteins comprised of Gα12 and Gα13 that are encoded by the GNA12 and GNA13 genes, respectively. G12 proteins themselves have also been found to be upregulated in many solid tumors, including gastric, prostate, breast and head and neck squamous cell cancers (HNSCC) [16–19]. Dominant-active forms of G12 proteins have been shown to induce transformation, migration, invasion and metastasis in many cell types [20]. Most of these effects are mediated via activation of Rho GTPase, although additional pathways such as NFκB, Hippo-YAP, and WNT-βCatenin have been implicated as well [21–25].

We recently showed that GNA13 is highly expressed in aggressive breast and prostate cancer cell lines, and that blocking GNA13 expression is sufficient to block cancer cell invasion [26, 27]. However, the impact of enhanced GNA13 activity on patient outcome and response to therapy remained unknown. In this study, we uncovered a crucial role of GNA13 in the acquisition of TIC-like phenotypes and therapeutic response in solid tumors, and found that GNA13 expression levels correlate with poor clinical outcomes in these cancers.

## Results

### GNA13 is a prognostic biomarker of survival and metastasis

**Fig. 1:**
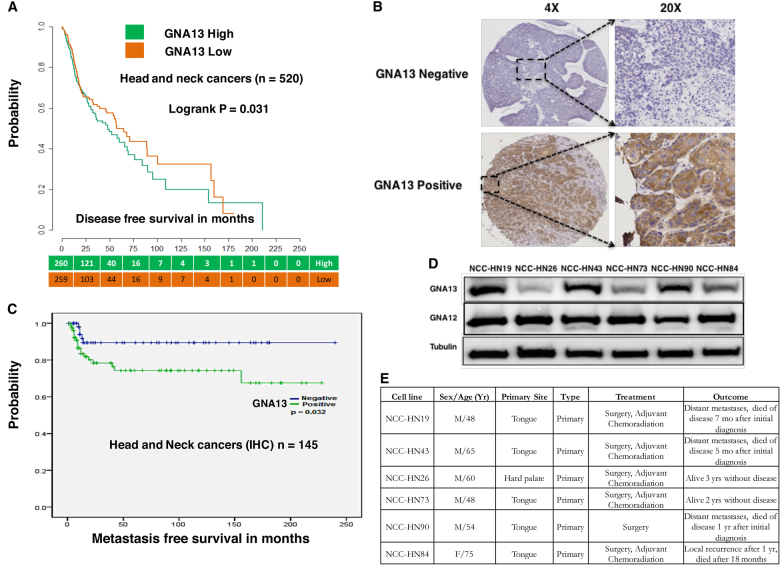
GNA13 is a prognostic marker of survival in solid tumors : Kaplan–Meier survival plot of **a** Head and neck cancer (HNSCC) patients comparing those expressing higher vs. lower than median GNA13 mRNA levels. The breakdown of number patients for High and Low GNA13 groups is indicated in the table below the graphs. **b** GNA13 staining of HNSCC tissue microarrays by immunohistochemistry (IHC) and a representative staining of sections negative (upper panel) and positive for GNA13 (lower panel). **c** Kaplan-Meier analysis showing distant metastases-free survival in patients that are positive vs. negative for GNA13 IHC staining. **d** Immunoblot for GNA12 and GNA13 in patient-derived HNSCC tumor cells. Tubulin is shown as loading control. **e** Table showing the names of the primary cell lines and clinical characteristics of the patient from which these cell lines were derived from

To assess the relationship between GNA13 expression levels and outcomes across solid tumors, we analyzed publically available expression data from TCGA (using cBioportal) [28] and KMPlot [29–31]. These analyses showed that tumors with high GNA13 mRNA expression were associated with poor survival in the head and neck (*p* = 0.031) (Fig. 1a and Supplementary Fig. 1 A) [29] cancers. A similar trend was observed with ovarian (*p* = 9.1 × 10^−5^) [32], lung (*p* = 2.5 × 10^−12^) [33] and gastric (*p* = 1.1 × 10^−7^) [34] cancers (Supplementary Fig. 1B-D). To validate these findings, we analyzed GNA13 protein expression by immunohistochemistry in 145 HNSCC tumor tissues, from patients that had no prior treatment (Fig. 1b). In all, 59% (*n* = 85) of tumors expressed high levels of GNA13 protein and this correlated with a significant reduction in distant metastases-free survival (*p* = 0.032) (Fig. 1c), but not loco-regional recurrence (data not shown). Immunoblot analysis of GNA13 protein levels of a panel of HNSCC patient-derived cell lines showed higher GNA13 expression in cell lines derived from tumors taken from patients that went on to develop distant metastasis (HN19, HN43, and HN90), with no variation seen in the ortholog GNA12 (Figs. 1d and e). Taken together, these data suggest that GNA13 expression is an indicator of poorer survival and progression to metastases for a range of solid tumors.

### GNA13 induces resistance to treatment with cytotoxic drugs and γ-Irradiation

**Fig. 2:**
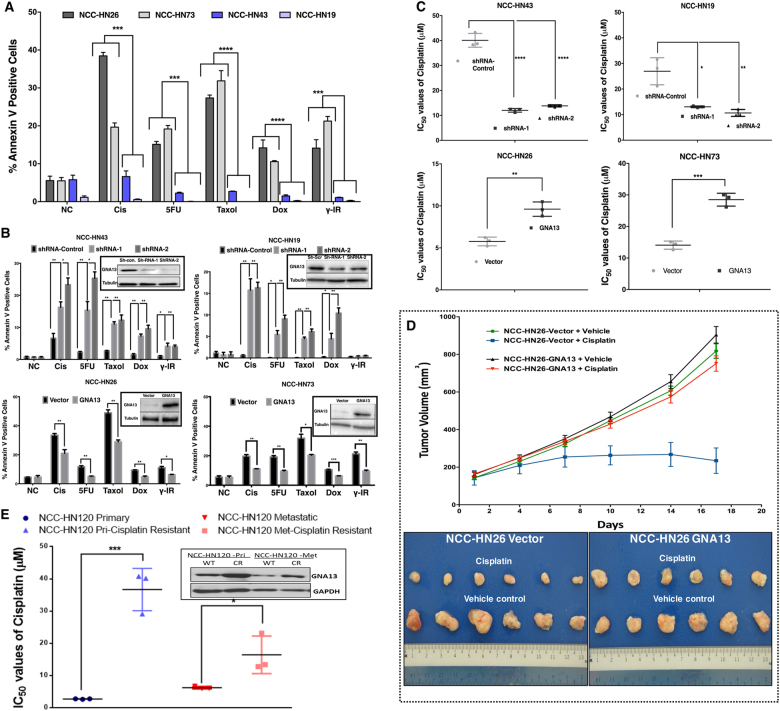
GNA13 induces resistance to treatment with cytotoxic drugs and *γ*-irradiation in HNSCC. **a** Basal GNA13 protein expression correlates to resistance to cytotoxic treatment-induced apoptosis in a panel of patient derived HNSCC cell lines. The cytotoxic treatment (*x*-axis)—induced apoptosis is shown as % Annexin V staining (*y*-axis) compared to untreated control (NC). See Fig.1E for the Immunoblot in the inset showings basal GNA13 expression.; Tubulin is used as control. **b** Knockdown of GNA13 in GNA13-high cells induces sensitivity while overexpression in GNA13-low cells induces resistance to cytotoxic treatment-induced apoptosis. Graphs show percentage of cells that underwent apoptosis (based on Annexin V staining (*y*-axis)) in response to cytotoxic treatment (*x*-axis) compared to negative controls (NC) after GNA13-knockdown in NCC-HN43 and NCC-HN19 and over-expression in NCC-HN26 and NCC-HN23 respectively. Immunoblot of GNA13 protein expression for each line is shown in the inset with tubulin as loading control. **c** Blocking GNA13 expression reduces the IC_50_ values to cisplatin, while overexpression does the opposite. Assessment of IC_50_ values from cell viability assays after cisplatin treatment in GNA13-knockdown (ShRNA-1 and ShRNA-2) NCC-HN43 and NCC-HN19 (compared to ShRNA-control), and GNA13-overexpression in NCC-HN26 and NCC-HN73 (compared to vector control) is shown. **d** GNA13 overexpression induces resistance to cisplatin-induced tumor regression in vivo. The volume of tumor xenografts in NOD/SCID mice using GNA13 overexpressing NCC-HN26 cells with/without cisplatin treatment, compared to vector control is shown (Top panel). Mice were treated with cisplatin (5 mg/Kg body weight) or vehicle control. Images of the harvested tumors after six doses of cisplatin or vehicle for NCC-HN26-vector and NCC-HN26-GNA13 expressing cells are shown (Lower panel). **e** GNA13 protein expression is higher in cisplatin resistant patient derived HNSCC cells. Assessment of IC_50_ values from cell viability assays after cisplatin treatment in cisplatin resistant (CR) NCC-HN120 cells compared to parental (WT) primary or metastatic lines is shown. Immunoblot for GNA13 is shown in the inset with tubulin as loading control. For all graphs, *p*-values denoted as: *, *p* < 0.05, **, *p* < 0.005, ***, *p* < 0.0005 and ****, *p* < 0.00005; error bars indicate standard deviation

To establish if the aggressive nature of tumors expressing high levels of GNA13 (GNA13-high) is related to poorer therapeutic response, we performed drug treatment on the panel of patient-derived HNSCC lines described above. The cells were treated with cisplatin, 5-fluorouracil (5-FU), paclitaxel, doxorubicin or *γ* **-**ionizing radiation (IR), all standard agents used in the management of HNSCC. Apoptosis assays showed that the GNA13-high cell lines NCC-HN43 and NCC-HN19 were resistant to all treatment modalities as compared to NCC-HN26 and NCC-HN73 cells that express lower levels of GNA13 (*p* < 0.0001) (Fig. 2a). Further, shRNA-mediated knockdown of GNA13 in NCC-HN43 and NCC-HN19 cells was sufficient to increase the sensitivity of these lines to cytotoxic or IR-mediated apoptosis (*p* < 0.01) (Fig. 2b). Conversely, over-expression of GNA13 in NCC-HN26 and NCC-HN73 (GNA13-low) cells induces resistance to the same treatments (*p* < 0.01) (Fig. 2b). Similar results were seen with response of the cells to increasing doses of cisplatin treatment (Supplementary Fig. 2A); NCC-HN43 and NCC-HN19 (GNA13-high) were more resistant to the treatment as compared to NCC-HN26 and NCC-HN73 (GNA13-low) cells (with IC_50_ values of 53.8 and 32.2 μM vs. 5.8 and 8.9 μM, respectively).

To directly assess the importance of GNA13 levels in the therapeutic sensitivities seen in the cancer cell lines, both loss- and gain-of-function approaches were undertaken. GNA13 knockdown in GNA13-high cells resulted in a 2–3-fold increase (*p* < 0.01) in their sensitivity to cisplatin, while its over-expression in GNA13-low cells increased IC_50_ values by 2-fold (*p* < 0.01) in these lines (Fig. 2c & Supplementary Fig. 2B). Furthermore, the increased sensitivity to cisplatin treatment upon loss of GNA13 is rescued by transfecting the shRNA-resistant GNA13 cDNA construct into two different GNA13-high cell lines (Supplementary Fig. 3A, B). Similar results were observed for treatment of the lines with manipulated GNA13 levels with doxorubicin (Supplementary Fig. 4A), paclitaxel and 5-FU (data not shown). To extend these findings into an in vivo setting, we established xenografts using NCC-HN26-vector or NCC-HN26-GNA13 cell lines and treated the mice with cisplatin. Evidently, xenografts from the GNA13-overexpressing line were resistant as compared to the vector control line (Fig. 2d). To further determine the importance of GNA13 expression level in an acquired drug resistance model, we established cisplatin-resistant (isogenic) lines from a pair of patient-derived lines from primary (NCC-HN120-Pri) and metastatic (NCC-HN120-Met) tumors (Fig. 2e, & Supplementary Fig. 4B) by treatment with escalating does of cisplatin and selection of survivors. Immunoblot analysis of GNA13 in these lines showed increased expression in the cisplatin-resistant cells as compared to isogenic (cisplatin-sensitive) parental cells (Fig. 2e). These data show that GNA13 level is an important component of the cytotoxic response of these cancer cells, and suggest that this protein plays an important role in intrinsic and acquired cisplatin-resistance in HNSCC.

### GNA13 promotes cancer stem cell-like properties in HNSCC cells

**Fig. 3:**
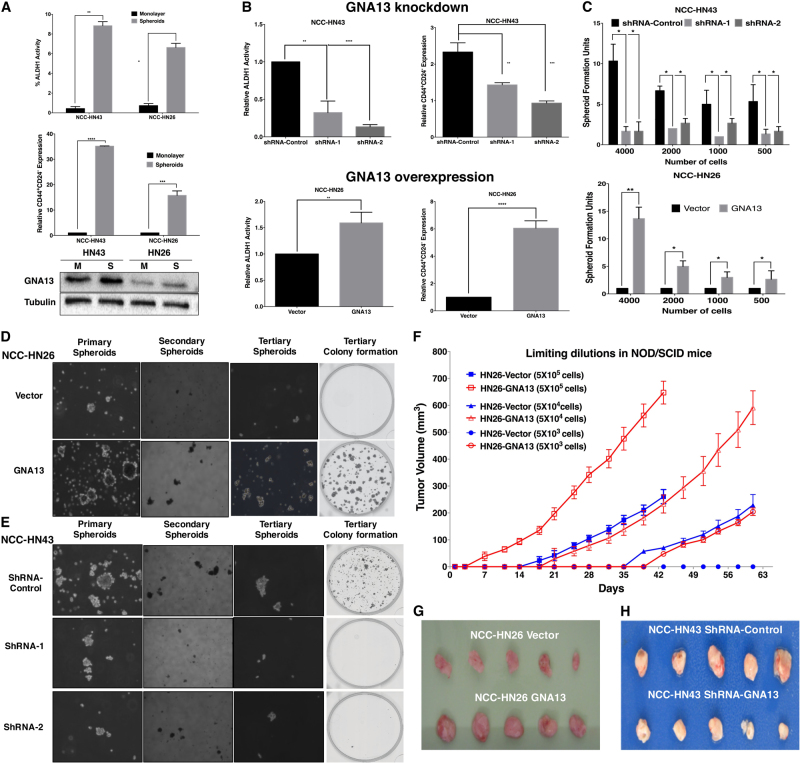
GNA13 promotes tumor initiating cell-like properties in HNSCC cells. **a** Cells grown in 3D spheroid cultures show increased TIC markers and GNA13 protein expression compared to cells grown in monolayers. Graphs showing TIC sub-population using ALDH1 activity and CD44^+^/CD24^−^-cell fractions in NCC-HN43 and NCC-HN26 grown as monolayers (M) and spheroids (S) (with Western blots showing GNA13 levels) **b** Blocking GNA13 in GNA13-high cells abrogates TIC markers while overexpression in GNA13-low cells induces them. Relative ALDH1 activity and relative CD44^+^/CD24^-^ is shown (*y*-axis) for GNA13-knockdown cells NCC-HN43 (ShRNA-1 and ShRNA-2, compared to ShRNA-control) (Top panel), and GNA13-overexpressing NCC-HN26 cells compared to vector control (Lower panel). **c** Knockdown of GNA13 suppresses the number of sphere forming units (SFU) in NCC-HN43 cells while overexpression in NCC-HN26 cells induces the SFUs. Number of sphere forming units is plotted in *y*-axis and the number of cells seeded in each well is shown in *x*-axis. **d**, **e** GNA13 is essential for long-term self-renewal of HNSCC cells. Cell culture images showing sphere formation of primary, secondary and tertiary spheroids in 3D culture, with subsequent colony formation in monolayer culture (Crystal violet stain) of (**d**) GNA13-overexpressing NCC-HN26 cells (compared to vector control) and (**e**) GNA13-knockdown in NCC-HN43 cells (ShRNA-1 and ShRNA-2, compared to ShRNA-control). **f** GNA13 is essential for early tumor initiation and induces tumor size. Graph showing tumor growth curves for limiting dilution experiments, where GNA13-overexpressing NCC-HN26 and vector control cells were injected into flanks of NOD/SCID mice (*n* = 5 each); Number of cells injected were: 5 × 10^5^, 5 × 10^4^ and 5 × 10^3^ and tumor volumes were measured for 61 days. Xenograft tumors harvested after 61 days of injecting 5 × 10^5^ cells in NOD/SCID mice is shown for **(g)** GNA13 overexpressing NCC-HN26 compared to vector control cells and (**h**) GNA13-knockdown NCC-HN43 cells (compared to ShRNA-controls). All experiments are performed at least three times with three replicates (*n* = 3) and a representative figure is shown. For all graphs, *p*-values denoted as: *, *p* < 0.05, **, *p* < 0.005, ***, *p* < 0.0005 and ****, *p* < 0.00005; error bars indicate standard deviation

We recently reported that expression profiling in control (GNA13-low) and GNA13-overexpressing MCF-10a breast epithelial cell lines show gene expression changes indicative of EMT upon elevation of GNA13 levels [35]. Additionally, analysis of expression profiles of the NCI-60 panel of cancer cell lines showed that GNA13 mRNA levels, but not GNA12, correlate significantly with the CSC/TIC marker ALDH1 (*p* = 0.037) (Supplementary Table 1). Given the significant overlap between EMT and TIC phenotypes, we assessed the relationship between GNA13 levels and the TIC state associated with drug resistance and metastasis. Spheroid cultures of NCC-HN43 and NCC-HN26 results in significant upregulation of TIC markers and higher levels of GNA13 when compared to cells grown in monolayer culture (Fig. 3a and Supplementary Fig. 5A and B) (*p* < 0.01).

To establish if GNA13 is necessary for the TIC-like phenotype, we measured ALDH1 activity and CD24^−^/CD44^+^ cell-fractions after GNA13 knockdown and overexpression (Fig. 3b). Knockdown in NCC-HN43 (GNA13-high) cells suppressed ALDH1 activity and CD24^-^/CD44^+^ cell-fractions while over-expression in NCC-HN26 (GNA13-low) cells had the converse effect (*p* < 0.01) (Fig. 3b). Transcript levels of stem cell markers KLF4, OCT4, SOX2 and Nanog corroborated these findings (Supplementary Fig. 5C), as did sphere formation assays, where GNA13 knockdown resulted in reduced sphere-forming units (SFUs) (Fig. 3c and Supplementary Fig. 5D), while over-expression enhanced SFU levels compared to their respective controls (*p* < 0.01) (Fig. 3c and Supplementary Fig. 4E).

One of the key characteristics of TICs is their ability to self-renew, measured by serial seeding of spheres in vitro. Consistent with the induction of TIC phenotype, the GNA13 over-expressing NCC-HN26 cells formed primary, secondary and tertiary spheres, and more colonies in adherent conditions compared to vector control cells (Fig. 3d), while GNA13 knockdown suppressed the ability of NCC-HN43 cells to form spheres or colonies in vitro (Fig. 3e). The “gold standard” test for TICs’ self-renewal and tumorigenicity is the increased ability to initiate tumors in vivo under limiting dilutions of the cells. To determine whether GNA13 induces TIC-like phenotype in vivo, we injected 5 × 10^5^, 5 × 10^4^, and 5 × 10^3^ NCC-HN26 cells stably expressing either the vector alone or GNA13 in NOD/SCID mice subcutaneously, and monitored tumor growth. NCC-HN26 cells expressing GNA13 initiated tumor growth significantly earlier as compared to vector control cells (Fig. 3f), leading to significant increase in tumor size at the end of 61 days (Fig. 3g). Most importantly, only GNA13 over-expressing cells could initiate tumors with 5 × 10^3^ cells while vector control cells completely failed to form tumors even after 61 days (Fig. 3f). In contrast, injection with 5 × 10^5^ NCC-HN43 cells that stably express either shRNA-control or shRNA-GNA13 in NOD/SCID mice showed a significant reduction in tumor sizes in GNA13-knockdowns as compared to controls (Fig. 3h). These data showed that GNA13 is necessary and sufficient for all properties associated with the TIC-state in HNSCC cells.

### GNA13 does not promote epithelial to mesenchymal transition in HNSCCs

**Fig. 4:**
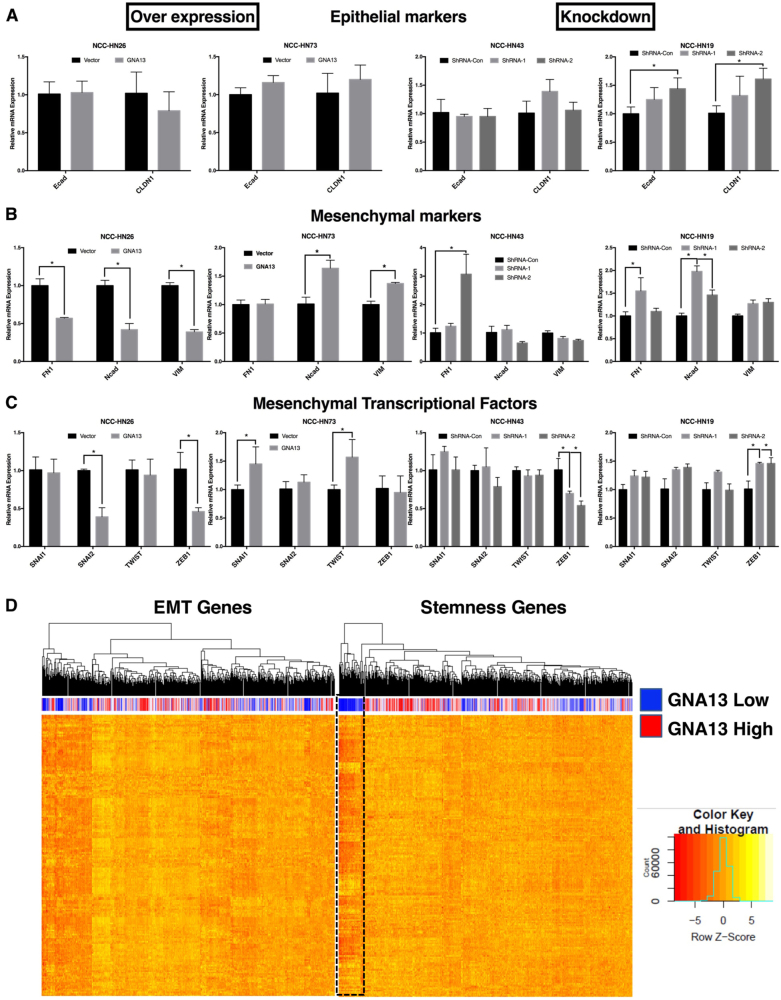
GNA13 does not induce Epithelial to Mesenchymal Transition (EMT) in HNSCC A real-time PCR screen for EMT marker expression in 4 different HNSCC cells either with enforced expression (left panel) or knockdown (right panel) of GNA13 showed no correlation of GNA13 expression to EMT induction. A real-time PCR analysis for **(a**) Epithelial markers—E-cadherin (Ecad) and Claudin-1 (CLDN1), **(b)** Mesenchymal markers—Fibronectin-1 (FN1), N-Cadherin (Ncad) and Vimentin (VIM) and **(c)** Mesenchymal Transcription Factors (TFs)—Snail-1 (SNAI1), Snail-2 (SNAI2), Twist-1 (TWIST) and Zinc Finger E-Box Binding Homeobox 1 (ZEB1) was performed in HN26 and HN73 (low-GNA13) cells stably expressing either vector control or GNA13, and in HN43 and HN19 (high-GNA13) cells stably expressing ShRNA-control or two different ShRNAs targeting GNA13. The mRNA expression of each gene is shown in y-axis as relative expression to either vector controls or ShRNA-controls respectively. **d** A hierarchical clustering analysis of GNA13 mRNA expression to established EMT markers or Stemness markers showed that GNA13 expression correlates to stemness rather than EMT inducing genes. The blue bar represents low GNA13 and the red bar represents high GNA13 expressing patients and the heat map shows EMT (left panel) or stemness (right panel) related genes’ expression as a heat map generated using raw z-scores from the same patients. For all graphs, *p*-values denoted as: *, *p* < 0.05, **, *p* < 0.005, ***, *p* < 0.0005 and ****, *p* < 0.00005; error bars indicate standard deviation

Recent studies suggest that drug resistance and metastasis could be induced by EMT in a number of different solid tumors [7]. Moreover, apart from the overlap in phenotype and transcriptional programs, it has also been shown that induction of EMT could lead to a TIC-like phenotype in breast epithelium [4]. Given the drug resistance and TIC phenotypes observed here, we sought to determine the contribution of EMT to the observed phenomena in HNSCCs. Real-time PCR analysis showed that there was no loss of epithelial markers such as E-cadherin or Claudin-1 (Fig. 4a), nor gain of expression of mesenchymal markers (Fig. 4b) or transcription factors (Fig. 4c) upon GNA13 overexpression. Similarly, GNA13 knockdown also had no effect on the same EMT markers (Figs. 4a–c). To validate this data using TCGA data for HNSCC, we examined tumors with high and low GNA13 expression (based on median expression levels), and ran unsupervised clustering for genes implicated in EMT or stemness (Fig. 4d). This data shows that high GNA13 expression correlated to genes implicated in stemness, but not EMT (Fig. 4d). These data indicate that the GNA13-induced drug resistance and TIC-like phenotypes are independent of EMT induction in head and neck cancer cells.

### GNA13 promotes TIC-like phenotype and drug resistance via NFκB and MAPK signaling pathways

**Fig. 5:**
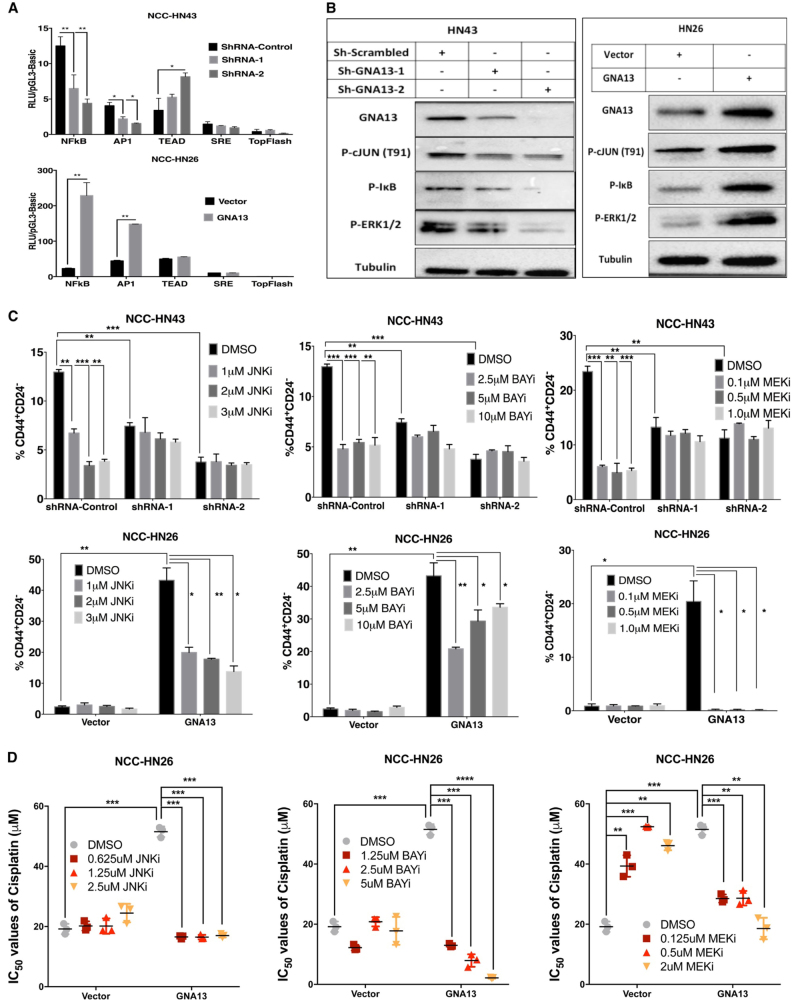
GNA13 promotes TIC-like phenotype and drug resistance via NFκB and MAPK signaling pathways. **a** GNA13 induces NFκB and AP-1 transcription factors’ activity in HNSCC cells. Graphs showing luciferase reporter activity driven by the following promoters: NFκB, AP-1, YAP/TAZ (TEAD), SRE and WNT-βCatenin (Top-Flash), in GNA13-knockdown NCC-HN43 (ShRNA-1 and ShRNA-2, compared to ShRNA-control), and GNA13 overexpressing NCC-HN26 compared to vector control cells. The ratio of relative light (RLU) units of the reporters to RLU of Renilla-Luciferase control is plotted as fold increase to pGL3-basic vector in *y*-axis. **b** GNA13 induces NFκB and JNK/MAPK/AP-1 signaling pathways. Immunoblots showing the effect of GNA13-knockdown or over-expression on phosphorylation of c-JUN, IκB and ERK1/2 in NCC-HN43 and NCC-HN26 cells (compared to ShRNA-Control or vector controls), respectively. Tubulin is shown as a loading control. **c** Blocking NFκB and/or JNK/MAPK/AP-1 signaling pathways abrogates GNA13-induced TIC markers. Graph showing effect of increasing amounts of JNK inhibitor SP6000125 (JNKi), NFκB inhibitor BAY-11-7082 (BAYi) and MEK1/2 inhibitor MEK162 (MEKi) on CD44^+^/CD24^−^—cell fractions in GNA13-knockdown NCC-HN43 (ShRNA-1 and ShRNA-2, compared to ShRNA-control) (top panel), and GNA13-overexpressing NCC-HN26 (compared to vector control) (lower panel). **d** Blocking NFκB and/or JNK/MAPK/AP-1 signaling pathways abrogates GNA13-induced Cisplatin resistance. Graph showing IC_50_ for cisplatin after blocking JNK (JNKi), NFκB (BAYi) or MEK1/2 (MEKi) pathways on GNA13 overexpressing NCC-HN26 cells compared to vector control. For all graphs, *p*-values denoted as: *, *p* < 0.05, **, *p* < 0.005, ***, *p* < 0.0005 and ****, *p* < 0.00005; error bars indicate standard deviation

To elucidate mechanisms that contribute to GNA13-induced TIC-like phenotype, we conducted a series of experiments using promoter-reporter assays downstream of GNA13: NFκB, AP-1, SRE, β-Catenin (TCF) and TEAD (YAP/TAZ) (Supplementary Fig. 6) [23, 24, 36, 37]. Screens in GNA13-knockdown NCC-HN43 cells showed NFκB and AP-1 activity were significantly suppressed (Fig. 5a), while GNA13-overexpression in NCC-HN26 cells induced both reporters compared to controls (Fig. 5a). These findings were supported by immunoblots of signaling pathways: reduced basal phosphorylation of c-Jun (JNK pathway), ERK1/2 (RAS-RAF-MAPK pathway) and IκB (NFκB pathway) after GNA13 knockdown (Fig. 5b), with the opposite effect in GNA13-overexpressing NCC-HN26 cells (Fig. 5b). To determine whether blocking either NFκB or AP-1 pathways reverses the GNA13-induced TIC phenotype, we measured the CD24^−^/CD44^+^ cell fractions after treating NCC-HN43 GNA13-knockdown cells with JNK inhibitor (JNKi, to block AP-1 activity), NFκB inhibitor (BAYi) or MEK inhibitor (MEKi, to block ERK1/2). Treatment with any of these inhibitors results in significant reduction in the CD24^-^/CD44^+^ cell fraction in NCC-HN43 control cells with high GNA13 levels (*p* < 0.01), but had no effect in GNA13-knockdown cells (Fig. 5c). In contrast, when GNA13 is overexpressed in NCC-HN26 cells, the CD24^-^/CD44^+^ cell fraction (Fig. 5c), ALDH1 activity (Supplementary Fig. 7A) and number of spheroids (Supplementary Figs. 7B and C) were suppressed by treatment with JNKi, BAYi or MEKi (*P* < 0.01), with MEKi showing the highest potency. Most importantly, treatment of NCC-HN26 cells with JNKi, BAYi or MEKi reversed cisplatin-resistance induced by GNA13, where IC_50_ values in GNA13 expressing NCC-HN26 cells were reduced to values similar to that of vector control cells (*p* < 0.001) (Fig. 5d and Supplementary Fig. 7D). Taken together, these data strongly support the notion that GNA13-induced TIC phenotype is mediated via activation of NFκB and JNK/MAPK-AP-1 signaling pathways, and that blocking these pathways can re-sensitize the HNSCC cells to cisplatin treatment.

## Discussion

The challenge for the field of cancer biology now that next generation sequencing is widely available, is to correlate genomics with cancer traits that confer an aggressive phenotype. In addition to making the necessary genotype-phenotype correlation, this information could allow targeting of relevant pathways in the context of individual tumors; this is the “sine qua non” of precision medicine. Indeed, current clinical trial strategies take the approach of grouping tumors of different contexts into “baskets” with similar driver genes. Several lines of evidence have implicated tumor cell properties such as stemness and EMT, with a range of detrimental outcomes, including the bane of cancer therapeutics, drug resistance and metastasis. It is increasingly becoming clear that, while a number of different signaling pathways can contribute to these phenotypes, identifying the relevant drivers may provide a vulnerability that can be exploited. In this regard, a number of GPCRs such as CXCR4, LPAR, PAR1, Angiotensin II (ANGII), Bradykinin, and S1PR, have been implicated in aggressive behaviors in a range of solid tumors [10, 20]. However, these pathways have been difficult to target due to a range of factors including overlapping and compensatory mechanisms [38]. Intriguingly, the majority of these GPCRs signal, at least in part, through the G12 proteins (GNA12 and GNA13), and we had hypothesized previously that targeting these G proteins or their downstream pathways may provide such a therapeutic vulnerability [20, 26, 27].

Analysis of cancer genomics databases show that GNA13 (but interestingly not GNA12) is indeed a strong prognostic marker for poor survival and metastasis in a range of solid tumors: head and neck, ovarian, lung and gastric [28, 30, 31]. A recent study confirmed these findings and showed that GNA13 expression is a potential biomarker for poor survival in gastric cancers [16]. From a more clinically practical perspective, we optimized IHC analysis of GNA13 and applied it across a panel of HNSCC tumors. The strong association with distant metastasis shown in this analysis further validates the notion that GNA13 levels can be used as a prognostic indicator of outcome, and demonstrated the value of GNA13 IHC to identify this patient subgroup.

Previous findings from our group and others have implicated elevated GNA13 expression with EMT in breast epithelial cells [35, 39]. Data presented here support the hypothesis that GNA13 over-expression resulted in treatment resistance while its silencing causes the opposite effect to a range of different cytotoxic therapies, including ionizing radiation. While one previous study had demonstrated a correlation between low GNA13 levels and sensitivity to Gemcitabine [40], our data suggest a more fundamental cancer trait that underlies an almost universal resistance to cytotoxic treatment. Consequently, we hypothesized that GNA13 might induce a tumor initiating or stem cell-like phenotype in these cancer cells. Indeed, we demonstrated through a limiting dilution xenograft assay that GNA13-induced a TIC/CSC-like phenotype in vitro and, importantly, in vivo. Surprisingly, we found that EMT was not a significant phenotype in the systems examined here nor in clinical data derived from TCGA for HNSCCs, although given the overlap between the two pathways (stemness vs. EMT), it is always difficult to dissect the two phenomena separately. In a recent report, Kuo et al. showed that Salinomycin suppressed the CSC/TIC growth, proliferation, invasion, CD44^+^/CD24^−^ expression and ALDH1 activity but had an opposite effect on EMT marker expression in HNSCCs, hence describing a scenario where the two observed phenomena have been uncoupled [41]. Data shown here indeed support this notion that GNA13 can induce tumor-initiating phenotype independent of EMT induction. Overall, these results support an emerging hypothesis that the EMT-CSC/TIC link is context-dependent and not necessarily interchangeable as a phenotype.

Targeting GNA13 by silencing its expression reversed the tumor initiation and drug resistance in HNSCC cancer cells and in tumors. However, applying this strategy in vivo would be challenging, and hence we explored downstream signaling pathways that mediate the phenotype described here. GNA13 activation leads to engagement of multiple downstream signaling pathways as shown in Supplementary Fig. 6 [23, 36]. Among them, the MAPK/AP-1 and NFκB pathways correlated significantly with GNA13 expression, and in our experiments the GNA13-induced TIC/CSC phenotypes. Previous studies have implicated AP-1 signaling in TIC/CSC-like phenotypes in colorectal cancer cells [42]. Similarly, activation of NFκB signaling has been shown to induce chemoresistance and TIC/CSC-like phenotypes in breast cancer cells [43, 44]. Although WNT-β-catenin [45] and Hippo-YAP [46] pathways have been independently implicated in CSC/TIC or EMT phenotypes, the activity of these signaling pathways did not correlate with the GNA13-driven TIC phenotypes described here. Crucially, our findings convincingly demonstrate that blocking GNA13-induced MAPK/AP-1 and/or NFκB signaling can indeed be a strategy to target TIC-induced tumor growth and therapeutic resistance in solid tumors that express high amounts of GNA13. However, how GNA13 induces drug resistance in the whole population while TICs/CSCs generally represent only a small subpopulation is not yet clear.

In conclusion, we show that GNA13 is a novel biomarker for prognosis and metastasis in solid tumors, and induces resistance to multiple cytotoxic modalities in HNSCCs. In addition, we show that GNA13 induces TIC-like phenotype in HNSCC cancer cells. Most importantly, reducing either GNA13 levels, or the activity of its downstream effectors MAPK/AP-1 or NFκB, can reverse the GNA13-induced TIC and drug-resistance phenotype. The theranostic implication of GNA13 suggests that a proportion of solid tumors could potentially be targeted through the strategy described here to potentially reverse an aggressive phenotype induced by GNA13-induced TICs.

## Materials and methods

### Cell lines and materials

Patient-derived HNSCC tumor cells were collected and processed as previously described [9, 47, 48]. Retroviral vector pMSCV-Blasticidine is a gift from Dr. Mathijs Voorhoeve (Duke-NUS Medical School, Singapore). GNA13 is cloned in pMSCV-Blast vector as described earlier [27]. Stable cell lines expressing either pMSCV-Vector or GNA13 are created as described earlier [27]. ShRNAs targeting GNA13 were cloned in modified pRetro-Super vector (gift from Dr. Mathijs Voorhoeve) and stable cell lines were generated using blasticidine selection as described [27]. RPMI or DMEM complete media with 10% FBS and 1% penicillin/streptomycin (GIBCO) is used to maintain the cell lines. For Western blots, antibodies used were as follows: Gα13 (ST1629, San Diego, CA, USA), α-tubulin (010M4813, Sigma, St Louis, MO, USA), p-cJun (Thr91, ab28853, Abcam, Cambridge, UK) total cJun (9165), p-ERK1/2 (Thr202/Tyr204, 9101), Total ERK1/2 (9102), and total IκB-alpha (4812) (Cell Signaling, Boston, MA, USA) and p-IκB-alpha (Ser32, Ma5-15087, Thermoscientific, Rockford, IL, USA). Gα13 antibody used for immunohistochemistry was purchased from Sigma (HPA010087).

### Preparation of cell lysates and western blots

Cells were seeded in 10 cm dishes and grown in the presence of fetal bovine serum (FBS) for 24 h followed by starvation in the presence of 0.1% FBS media overnight (for signaling pathway analysis only). Fresh 10% FBS was added and signaling was stimulated for 15–20 min. Cells were then washed with phosphate-buffered saline and lysed in protein extraction buffer (50 M HEPES pH 7.5, 1 mM EDTA, 3 mM dithiothreitol, 10 mM MgSO_4,_ 1% polyoxyethylene-10-lauryl ether) with phosphatase and protease inhibitors. BCA (Thermo Scientific) kit was used to determine the protein concentrations of the lysates. Aliquots (10–20 μg) were separated on a 10% SDS-PAGE and transferred to PVDF membrane. The membrane was incubated with the specific primary antibodies overnight in 4°C followed by incubation with respective secondary antibody linked to horseradish peroxidase (Millipore, San Diego, CA, USA), and visualized by chemiluminescence (ECL, Thermo Scientific).

### RNA analysis

Total RNA extraction and reverse transcription was performed as previously described [49]. Real-time PCR of cDNA was performed using iTaq Universal SYBR Green Supermix (Bio-Rad Laboratories, Hercules, CA, USA) and a CFX96 Real-time PCR system (Bio-Rad Laboratories) according to the manufacturer’s instructions. Reactions were carried out in triplicate with Actin or HPRT served as the normalizing controls. Primer sequences used were described previously [9, 27].

### Annexin V apoptosis assay

Tissue culture conditions and drug treatments were performed as described [49]. 5-Fluorouracil, cisplatin, paclitaxel and doxorubicin were purchased from Sigma-Aldrich (St. Louis, MO, USA). 1 × 10^5^ Cells were seeded in six well plates followed by treatment with 10 μM Cisplatin, 300 μM 5-Fluorouracil, 0.05 μM Paclitaxel and 0.345 μM doxorubicin for 72 h. For γ-Irradiation, the cells were treated once with 15 Gy of ɣ-radiation using Gamma cell 40 exactor (Best Theratronics, Ontario, Canada) and incubated for 72 h. Annexin V staining was performed as recommended by manufacturer (Life Technologies, Netherlands). The samples were read out using FACSCanto II (BD Biosciences, San Jose, CA, USA) and analyzed with FACSDiva software (BD Biosciences) within 4 h.

### Cell proliferation assay and determination of IC_50_ values

Cells were seeded in complete growth medium at a density of 2000–4000 cells/well in 96-well tissue culture plates based on their cell doubling times. Cells were pre-treated with Jun kinase inhibitor (JNKi) SP600125, NFκB inhibitor (BAYi) BAY-11-7082 (Sigma) or MEK1/2 inhibitor (MEKi) MEK162 (Selleckchem, Houston, TX, USA) for 24 h. After serial dilutions, 100 μl of complete growth medium containing 5-Fluorouracil, cisplatin, paclitaxel or doxorubicin were added to cells in increasing amounts. DMSO was used as controls. Plates were incubated for 72 h after which cell viability was assessed by CellTitre-Glo^®^ Luminescent Assay according to the manufacturer’s instructions (Promega, Madison, WI, USA). Reactions were carried out in triplicate and analyzed with GraphPad Prism 6 (GraphPad Software Inc., San Diego, CA, USA).

### Spheres formation and colony formation assay

Tumor spheres were formed as described previously [9]. 60 mm Petri (ultra-low adherent) dishes were used (Grenier Bio-One, Kremsmünster, Austria) for self-renewal assays or GravityPLUS™ Hanging Drop plates (Insphero, Brunswick, ME, USA) were used for sphere formation alone. The primary spheres were measured after 4 days and reseeded as single cells for secondary sphere formation for 6 days and reseeded again for tertiary sphere formation for 9 days. All the spheres were grown in special spheroid culture media as described previously [9]. For colony forming assays, tertiary tumor spheres were trypsinized into single cells and re-plated onto 6-well plates in RPMI medium supplemented with 10% FBS and 1% penicillin-streptomycin. The cells were then grown in standard culture conditions for 21 days with fresh media replaced every 3 days. The colonies were stained with crystal violet (0.25% w/v) (Sigma-Aldrich), and the clone numbers were counted using a stereomicroscope.

### ALDEFLUOR assay and flow cytometry analysis for CD24 and CD44 expression

ALDH1 activity was measured using the ALDEFLUOR assay kit (StemCell Technologies Inc., British Columbia, Canada) following the protocol as described previously [9]. For CD44^+^/CD24^-^ expression, cells were stained with PE-conjugated CD44 antibody and FITC-conjugated CD24 antibody (BD Biosciences) according to manufacturer’s protocol. In brief, trypsinized cells were resuspended in staining buffer containing PE-CD44 and FITC-CD24 antibody and incubated for 30 min at room temperature. The cells were sorted using flow cytometry (FACS) analysis (BD Biosciences). Reactions were performed in triplicates.

### Reporter assays

A total of 1 × 10^5^ cells were plated in 6-well plates for 24 h. Then, cells were transiently transfected with 1μg of 4X-AP-1-Luciferase reporter or 6X-NF-κB-Luciferase reporter (gifts from Prof. Doris Mayer, Germany) [50], SRE-Luciferase (gift from Dr. Ted Meigs, North Carolina) [24], TEAD-Luciferase (gift from Dr. Mathijs Voorhoeve) [51] or TOP-FLASH-luciferase (gift from Prof. David Virshup) using Lipofectamine (Invitrogen, Germany). A promoter-less pGL3-Basic was used as a negative control. Reporter luciferase activity was measured in relative light units 24 h post-transfection using the Dual Luciferase Assay System (Promega). pGL3-Renilla luciferase (100ng/well) was used as an internal control. The ratio of relative light units from firefly luciferase activity from the reporters to relative light units from Renilla luciferase was calculated and fold change to pGL3-Basic was plotted.

### PDX and cisplatin treatment studies

All the procedures were approved and carried out in accordance with the guiding ethical principles of the SingHealth Centralised Institutional Review Board (CIRB 2007/441/B). Animal studies were carried out in accordance with animal care and use guidelines approved by Biological Resource Centre, Singapore (IACUC number 161111). For limiting dilution experiments, 5 × 10^5^, 5 × 10^4^ or 5 × 10^3^ NCC-HN26 cells expressing either vector alone or GNA13 were injected subcutaneously in the lower flank of the NOD-SCID mice (In vivos, Singapore) (*n* = 5) respectively. All mice were monitored for tumor growth at the site of inoculation and tumor volumes were measured twice a week using Vernier caliper for 61 days or till the tumor volume reaches 600 mm^3^ whichever is earlier. The tumor volume was calculated using the following formula V = a × b^2^ × 0.52, where “a” is the largest and “b” the smallest diameter of the tumor.

To determine the effect of cisplatin, mice were injected with NCC-HN26-vector (5 × 10^6^) or GNA13 (3 × 10^6^) (*n* = 10) in two independent experiments. These were monitored for tumor growth twice a week using Vernier caliper as mentioned above. When the tumor volume reached approximately 100–200 mm^3^, the animals were randomized into 4 different groups: Group-1 vehicle control (*n* = 10) injected with NCC-HN26-vector (5 × 10^6^); Group-2 cisplatin (5 mg/kg, Q4DX3, i.p.) (*n* = 10) injected with NCC-HN26-vector (5 × 10^6^); Group-3 vehicle control (*n* = 10) injected with NCC-HN26-GNA13 (5 × 10^6^); Group-4 cisplatin (5 mg/kg, Q4DX3, i.p.) (*n* = 10) injected with NCC-HN26-GNA13 (5 × 10^6^). Body weight was measured every day and tumor volumes were monitored twice a week as mentioned above. At the end of the treatment the tumors from the animals were excised and fixed in 10% Neutral buffered formalin for immunohistochemical analysis. 4 animals died in each sample group and were excluded from the final analysis. Sample size for both animal experiments was calculated using ANOVA, where E (Degree of variance) = (Samples per group X number of groups)—number groups. E of 20-25 was considered optimal for these experiments. The groups were randomized based on body weight correcting for variation in body weight.

### Kaplan–Meier survival analysis using TCGA RNA seq database

RNA-seq data was downloaded from The Cancer Genome Atlas (TCGA) for head and neck squamous cell carcinoma (HNSC, *n* = 520). Samples were stratified to low and high expression of GNA13 based on median expression of the gene, and compared for survival and stemness or EMT. Individual gene signatures were selected from the molecular signatures database (MSigDB) portal, genes representing stemness (MSigDB ref: M9473) and EMT (MSigDB ref: M5930) were used. The expression of genes in each of these signatures was displayed as a heatmap. Overall survival was compared between groups and a boxplot used to visualize the distribution of GNA13 expression in each group. All data analysis was carried out using R 3.2.2 (R: A Language and Environment for Statistical Computing. R Core Team. 2016 https://www.R-project.org). TCGA RNA-seq data was downloading using the RTCGA package (Marcin Kosinski and Przemyslaw Biecek (2016). RTCGA: The Cancer Genome Atlas Data Integration. R package version 1.1.14.) and converted to Log2-CPM (counts per million) utilizing the voom function in the limma package as described in [52]. Survival curves were compared using the Mantel–Haenszel test.

### Statistical analysis

All in vitro experiments were performed in triplicate in three biological replicates. Statistical analysis was done using GraphPad Prism 6. Values were expressed as mean ± SEM. Differences/correlations between groups were calculated with Student’s *t*-test. A *p*-value < 0.05 was defined as significant. Survival outcome analyses for HNSCC IHC data (*n* = 145) were performed using PASW Statistics (Version 18.0, IBM, Armonk, NY, USA), using Kaplan-Meier analysis and a log-rank test was used to compare between cohorts with different expression levels of GNA13. The sample size was calculated using power analysis using expected high GNA13 of 70% and low GNA13 of 70% based on Western blot screening for GNA13 protein previously done in the lab in HNSCC primary cell lines. A *p*-value of less than 0.05 was deemed significant. A detailed description of Kaplan-Meier analysis performed online using kmplot for ovarian, lung and gastric cancers is given in supplementary methods.

## Electronic supplementary material

Supplementary material
