# Supplementary material for: GNA13 expression promotes drug resistance and tumor-initiating phenotypes in squamous cell cancers
Source: Oncogene. 2017 Dec 19;37(10):1340–53. doi: 10.1038/s41388-017-0038-6 (PMC6168473; doi:10.1038/s41388-017-0038-6)
Supplement: Supplementary file 1 — Supplementary material [file 41388_2017_38_MOESM1_ESM.docx]

**Supplementary Materials:**

**Supplementary methods**

***Immunohistochemistry***

Tissue micro array (TMA) samples of 195 head and neck cancer patients diagnosed at the National Cancer Centre, Singapore from Jan 1990 to March 2011 were analyzed for GNA13 expression using immunohistochemistry (IHC). TMA tissue sections were stained for Gα13 (GNA13) protein expression in a Bond Max Automated Immunohistochemistry staining system (Leica Microsystems GmbH, Germany) according to the following protocol. After deparaffinisation, antigen-retrieval was performed using Leica Bond^TM^ Epitope retrieval solution (pH6). After washing steps, endogenous peroxidase was blocked for about 15 mins in 3% H_2_O_2_. Then, the tissues were treated with 10% goat serum for 30 mins to block the non-specific reactions. Subsequently, the tissues were incubated overnight with primary antibody, anti-Gα13 (Cat# HPA010087, Sigma) at the dilution of 1:500 at 4° C. Then, the slides were incubated with secondary antibody (BondTM anti-rabbit IgG Polymer) for 5 mins. The slides were then rinsed with Bond^TM^ Wash Solution and treated with DAB-Chromogen detection reagent (Bond^TM^ Refine Detection Kit, Leica Microsystems GmbH, Germany) for 7 mins. Finally, the slides were counter stained with hematoxylin for 5 mins, re-hydrated and mounted in synthetic mounting media.

Two independent experienced pathologists (JSH, KHL) scored the staining intensity. Clinical data was available through the institutional database. Patients with prior history of cancer were excluded from the study as they might have received limited treatment. The study was approved by the Singhealth Centralized Institutional Review Board (CIRB 2007/441/B) and informed consent form all the patients was obtained. The tissues sections with a staining score of 0 are considered negative and rest of the tissues were considered positive and used for survival analysis.

For Ki67 staining, a similar protocol was followed and primary antibody (cat# ab15580) at the dilution of 1:1000 was used. For negative controls, BondTM Antibody Diluent was used instead of the primary antibody.

***Kaplan-Meier survival analysis using kmplot.com for ovarian lung and gastric cancer***

The survival analysis for patients expressing higher GNA13 mRNA expression (higher than the median GNA13 mRNA) versus patients with lower GNA13 mRNA expression (lower than the median) was auto generated using kmplot.org using the following parameters:

| **Cancer type** | **Affymetrix probes of GNA13 mRNA used for analysis** | **Total number of patients** | **Expression range of the GNA13 probes** | **Median GNA13 mRNA**  **(Cutoff value )** | **GNA13 high** | **GNA13 Low** |
| --- | --- | --- | --- | --- | --- | --- |
| Ovarian cancer | 206917_at  211611_s_at  216208_s_at  203168_at  224761_at  227539_at | 1648 | 149 - 2564 | 539.33 | 389 | 158 |
| Lung cancer | 206917_at | 2437 | 1 - 1268 | 60 | 1960 | 1061 |
| Gastric cancer | 206917_at | 1222 | 1 - 576 | 79 | 674 | 548 |
|  |  |  |  |  |  |  |

Supplementary Figures:

**
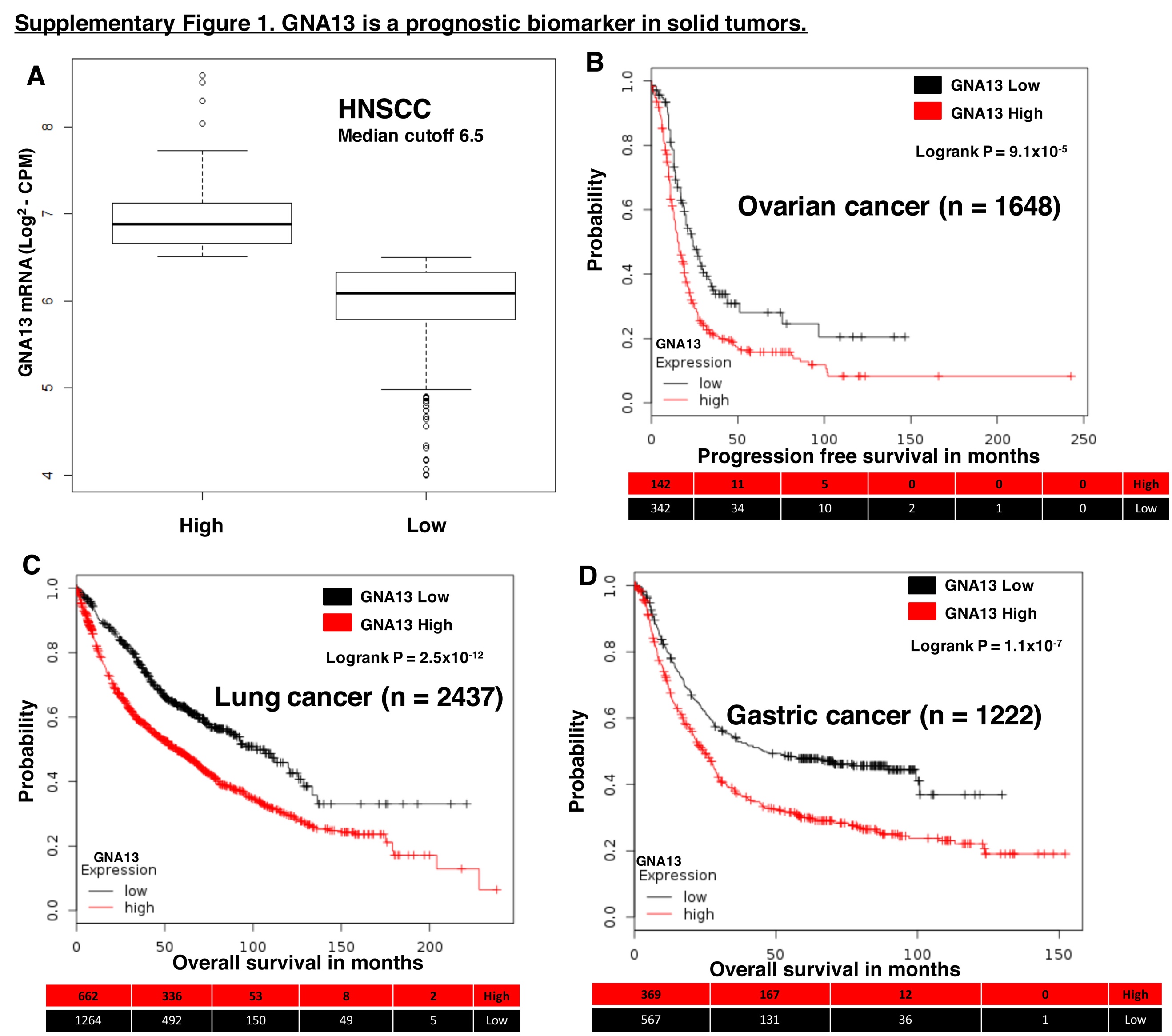
Supplementary Figure 1. GNA13 is a prognostic biomarker in solid tumors:** (A) The box plots represent the patient stratification based on GNA13 median expression as GNA13- low and -high that was used for survival analysis in Fig.1A for HNSCC (median cutoff 6.5). The GNA13 mRNA expression is shown in the graph as Log^2^ count per million (CPM). (B, C, D) Kaplan-Meier curves for survival was performed after stratifying patients with high and low GNA13 median mRNA expression in (B) Ovarian, (C) Lung and (D) gastric cancers. The probability is plotted in y-axis against progression free/overall survival in months in x-axis.

**
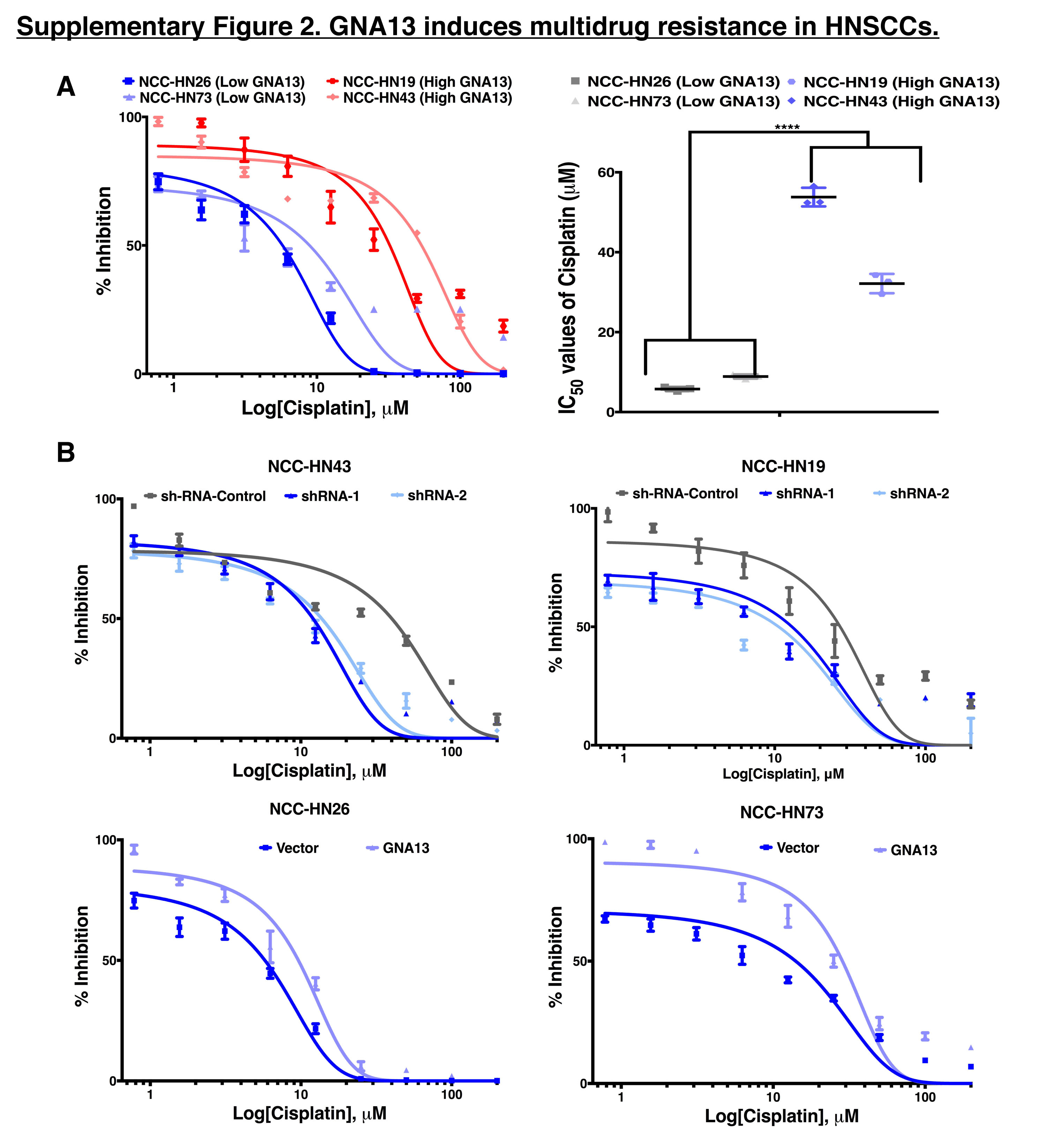
Supplementary Figure 2. GNA13 induces multidrug resistance in HNSCCs:** (A) Basal GNA13 expression correlates to resistance to cisplatin treatment in head and neck cancer cells. Dose response curves were generated using cell viability assays post-treatment with increasing dose of cisplatin in 4 different primary head neck cancer cell lines. Two of them had higher GNA13 expression (in red) and two others had lower GNA13 expression (in blue). Inhibition of cell viability as % of DMSO treated cells was calculated and plotted in y-axis against cisplatin concentration (Log(μM)) in x-axis (Left panel). IC_50_ values calculated using the dose response curves and are plotted in y-axis in μM (right panel). (B) Knockdown of GNA13 expression suppressed the IC_50_ values for cisplatin treatment in two different head and neck cancer cells NCC-HN43 and NCC-HN19 cells (Upper panel). Enforced expression of GNA13 induced IC_50_ values for cisplatin treatment in NCC-HN26 and NCC-HN73 cells (Lower panel). In all 4 curves % inhibition is plotted in y-axis and concentration of cisplatin is indicated in Log μM in x-axis.

**
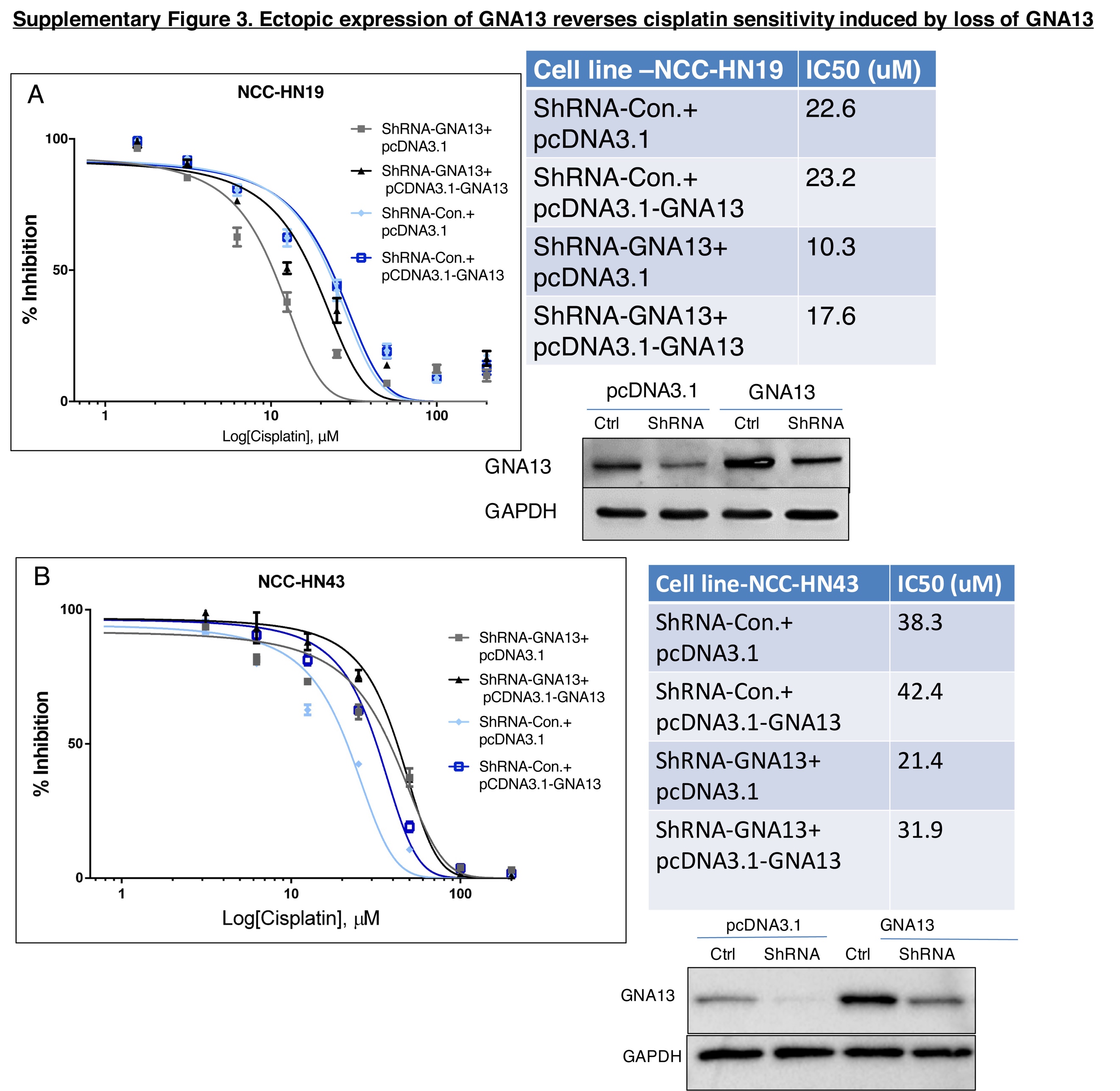
Supplementary Figure 3. Ectopic expression of GNA13 reverses cisplatin sensitivity induced by loss of GNA13:** Knockdown of GNA13 in (A) NCC-HN19 and (B) NCC-HN43 sensitized these cells to cisplatin. This effect was rescued by ectopic overexpression of shRNA-resistant GNA13 construct in the knockdown cells. The tables show the respective IC^50^ values for each condition and immunoblots show GNA13 protein expression.

**
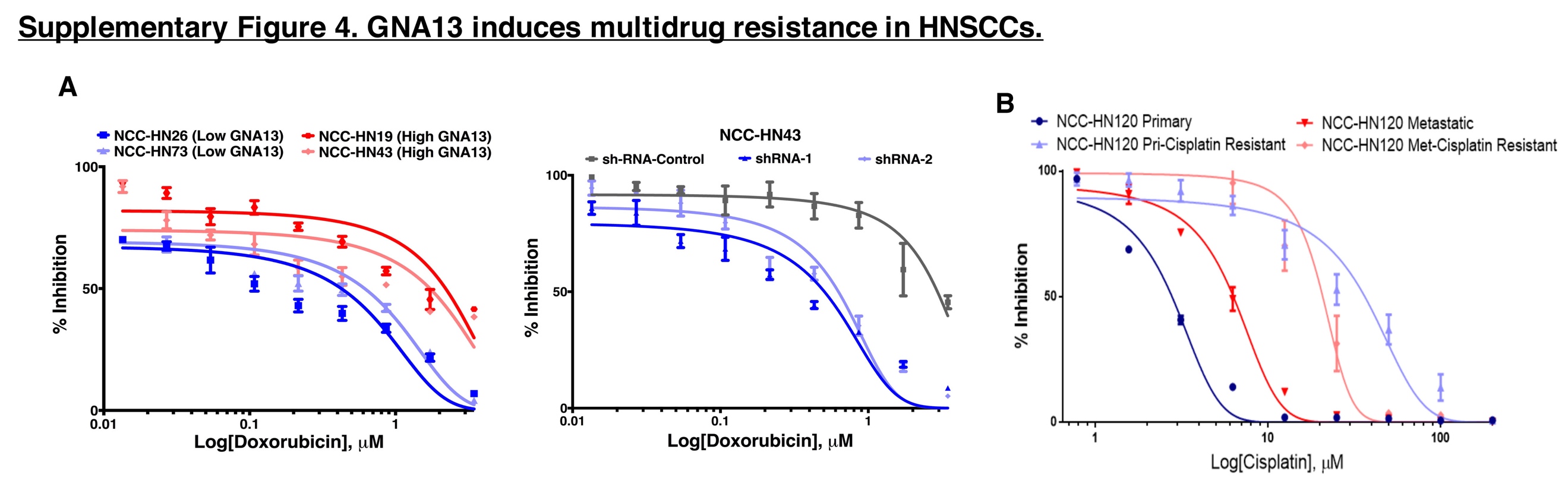
Supplementary Figure 4. GNA13 induces multidrug resistance in HNSCCs:** (A) GNA13 expression correlates to resistance to doxorubicin treatment in a panel of head and neck cancer cells (Left panel). Knockdown of GNA13 in NCC-HN43 cells suppresses the IC_50_ values for doxorubicin treatment. The graphs show dose response curves with % inhibition of cell viability in y-axis and concentration of doxorubicin in log μM in x-axis (Right panel). (B) Cisplatin resistant correlates to GNA13 expression in head and neck cancer patient derived cells NCC-HN120 from primary and metastasis sites (see GNA13 expression in Fig.2E). Cisplatin resistant cells HN120-primary and metastasis-derived cells (in red) were generated using the parental lines (in blue) after growing them in cisplatin for 2 months. The dose response curves in the graph shows % inhibition of cell viability in y-axis and concentration of cisplatin used in Log μM in x-axis

**
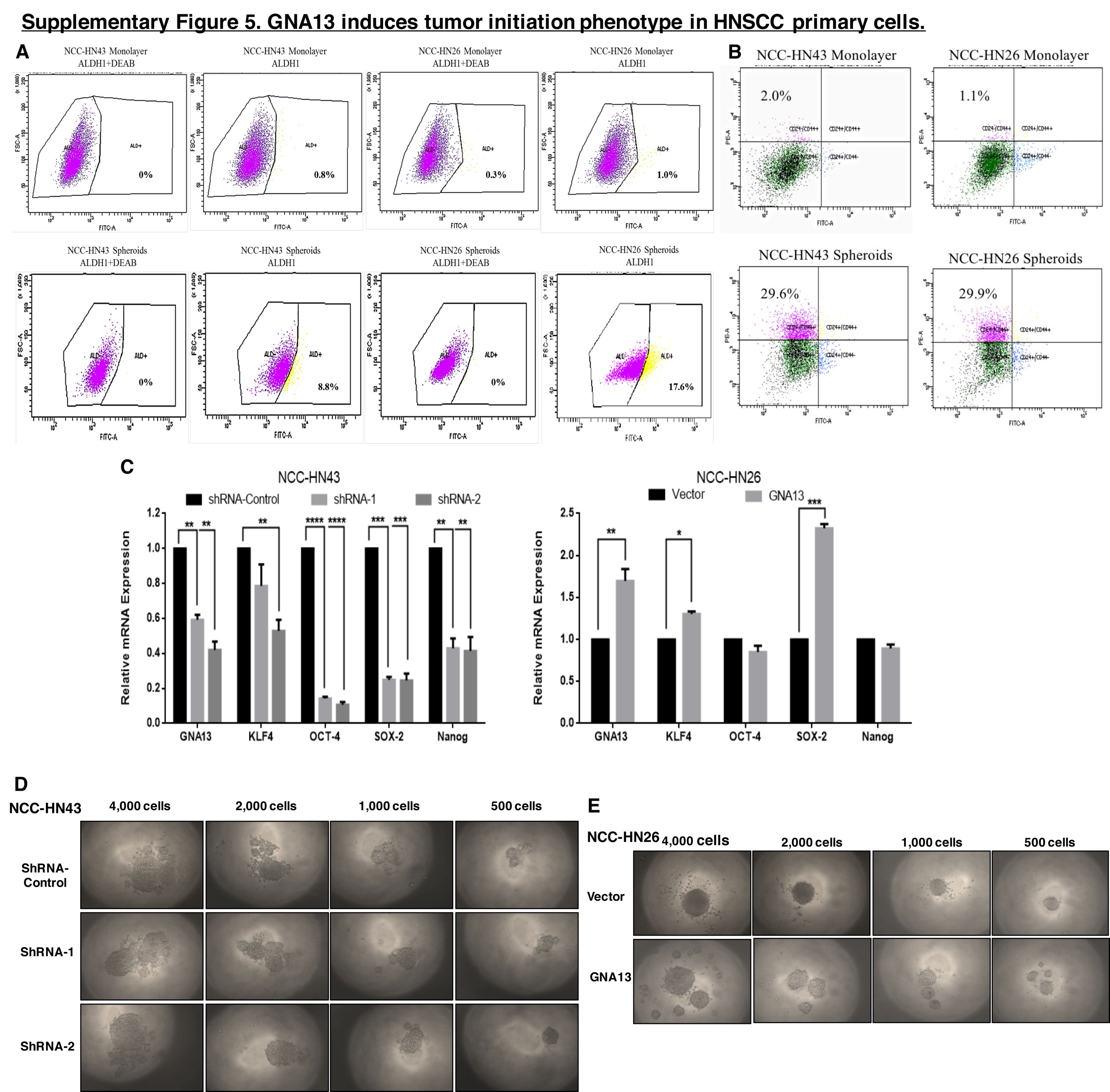
Supplementary Figure 5. GNA13 induces TIC like phenotype in HNSCC primary cells:** (A, B) NCC-HN43 and NCC-HN26 cells grown in 3D spheroids are marked by higher GNA13 protein expression and TIC markers when compared to cells grown in monolayers. (A) A representative analysis dot blot for ALDH1 activity using Aldefluor assay is shown for NCC-HN43 cells and NCC-HN26 cells grown either in monolayer (top row) or spheroids (bottom row). Gating was done using respective cells treated with DEAB (an inhibitor of ALDH1 activity), considering all cells treated with DEAB as background and marked as ALDH1 negative (ALD-). The green fluorescence induced by ALDH1 activity is measured and plotted against FSC-Area. The percentages of ALDH+ cells are shown in the plot (B). NCC-HN43 and NCC-HN26 cells grown in 3D spheroid culture had higher expression of the TIC marker CD24^-^ /CD44^+^. Cells were fixed and stained with antibody against CD24 conjugated to FITC and CD44 conjugated to PE. The cells were sorted using FACS analysis and the cells positive for PE is plotted in y-axis and cells positive for FITC plotted in the x-axis as shown in the figure. Cells positive CD44 and negative for CD24 cells (as %) are on the left top square as indicated. (C) Knockdown of GNA13 using two different shRNAs against GNA13 suppressed the mRNA expression (y-axis) of stemness markers KLF4, OCT-4, SOX-2 and Nanog in NCC-HN43 cells. Enforced expression of GNA13 induces the expression of stemness markers KLF4, OCT4, SOX-2 and Nanog. Relative mRNA expression is shown in y-axis (relative to shRNA control in NCC-HN43 and vector control in NCC-HN26 cells respectively) and the name of the gene in x-axis including GNA13. (D) Knockdown of GNA13 expression using shRNAs abrogates sphere forming units in NCC-HN43 cells compared to ShRNA-control cells. (E) Enforced expression of GNA13 induced the number of sphere forming units in NCC-HN26 cells compared to vector control cells. The indicated number of cells were seeded in a hanging drop 96 well plate model. The pictures of the spheroids were taken after 48 h and a representative picture is shown (E&F).

**
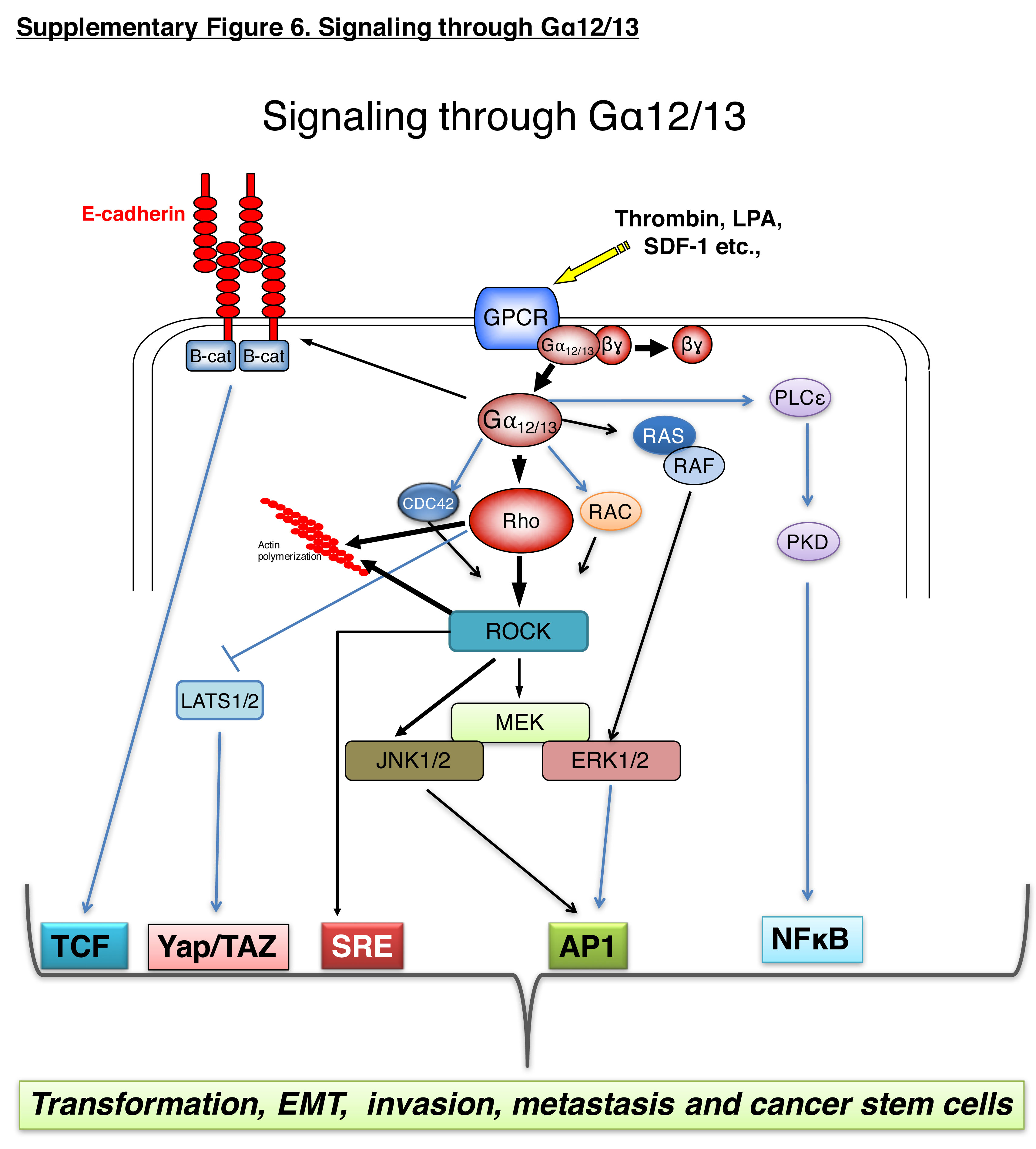
Supplementary Figure 6. Signaling through Gα12/13:**

A schematic representation of G12 protein signaling and its impact on tumor biology based on previous publications (See references 35-39).

**
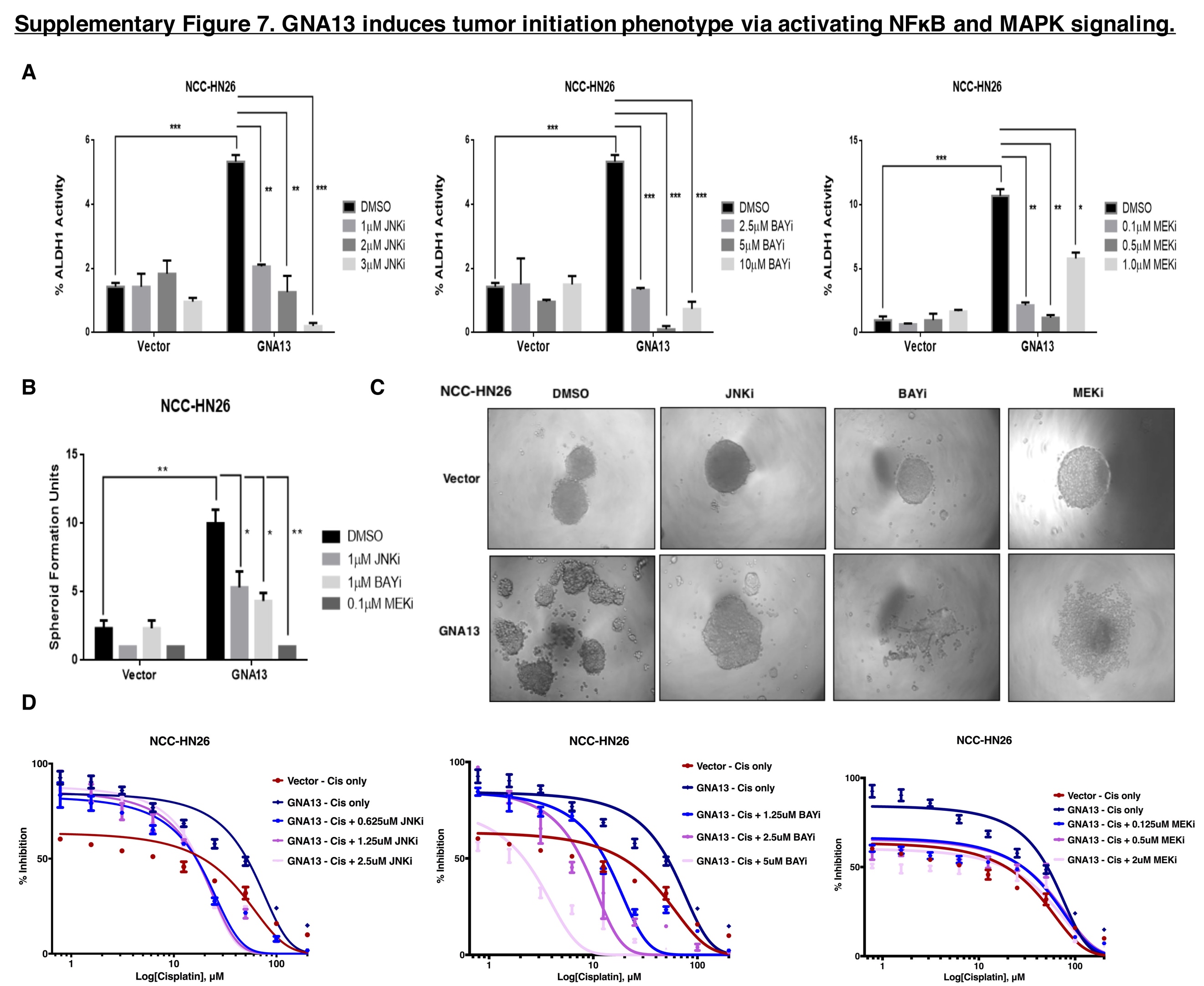
Supplementary Figure 7. GNA13 induces TIC-like phenotype via activating NFκB and MAPK signaling:** (A) In NCC-HN26 cells, blocking either JNK or NFκB or MEK1/2 signaling using increasing amounts of specific inhibitors of JNK (SP6000125, 1-3μM) or NFκB (BAY11-7082, 2.5-10μM) or MEK1/2 (MEK162, 0.1-1μM) inhibits GNA13 induced TIC/CSC marker ALDH1 activity (y-axis) when compared to DMSO alone treated cells. (B) In NCC-HN26 cells, blocking either JNK or NFκB or MEK1/2 signaling using specific inhibitors in amounts as indicated suppressed the number of GNA13-induced sphere forming units in 3D culture . (C) Representative cell culture images showing effect of JNKi, BAYi and MEKi on sphere formation in GNA13 overexpressing NCC-HN26 cells compared to vector control. (D) GNA13-induced cisplatin resistance is suppressed by blocking JNK or NFκB or MEK1/2 signaling pathways. Cell viability is measured with the increasing amounts of cisplatin, in the presence of increasing amounts of JNKi or BAYi and MEKi as indicated. % inhibition is plotted in y-axis against the concentration of cisplatin in Log μM in x-axis.

**Supplementary Table 1. GNA13 mRNA expression positively correlates to mRNA expression of TIC/CSC marker ALDH1 in NCI-60 cancer cell line panel:**

|  | GNA12 vs GNA13 | **GNA13 vs ALDH1** | GNA12 vs ALDH1 |
| --- | --- | --- | --- |
| **Pearson R** | -0.094662688 | 0.193321977 | -0.08834201 |
| **p Value** | ns | 0.0374 | ns |
| **GNA12** | **GNA13** | **ALDH1A1** | **NCI-60 Cell name** |
| 93.3 | 22.85 | 172 | 786-0 |
| 77.9 | 15.85 | 1190 | A498 |
| 14.9 | 36.85 | 6030 | A549/ATCC |
| 70.3 | 13.295 | 1970 | ACHN |
| 13 | 14.455 | 2.51 | BT-549 |
| 50.8 | 33.2 | 922 | CAKI-1 |
| 4.91 | 25.3 | 11.7 | CCRF-CEM |
| 11.4 | 12 | 663 | COLO 205 |
| 80.5 | 15.725 | 2.72 | DU-145 |
| 48.3 | 25.35 | 970 | EKVX |
| 11.2 | 16.25 | 788 | HCC-2998 |
| 10.1 | 21.45 | 487 | HCT-116 |
| 11.1 | 33.5 | 9.23 | HCT-15 |
| 5.72 | 19.55 | 3.21 | HL-60(TB) |
| 57.2 | 22.3 | 13.5 | HOP-62 |
| 65.7 | 55.05 | 3.85 | HOP-92 |
| 35.1 | 15.94 | 5.59 | HS 578T |
| 32.1 | 20.1 | 1070 | HT29 |
| 46.1 | 21.2 | 483 | IGROV1 |
| 13.7 | 18.25 | 81.8 | K-562 |
| 15.7 | 24.75 | 889 | KM12 |
| 32.5 | 29.95 | 5.48 | LOX IMVI |
| 6.02 | 30.95 | 39.4 | M14 |
| 18.4 | 46.35 | 71.7 | MALME-3M |
| 5.59 | 16.925 | 8.46 | MCF7 |
| 8.4 | 12.41 | 12.3 | MDA-MB-231/ATCC |
| 6.48 | 19.85 | 212 | MDA-MB-435 |
| 5.02 | 23 | 2.54 | MOLT-4 |
| 71.3 | 33.35 | 4.62 | NCI-H226 |
| 30.6 | 32.25 | 3.63 | NCI-H23 |
| 24.5 | 29.4 | 8.51 | NCI-H322M |
| 20 | 26.75 | 762 | NCI-H460 |
| 16.2 | 44.2 | 4500 | NCI-H522 |
| 26.8 | 8.19 | 5.22 | NCI/ADR-RES |
| 9.99 | 23.7 | 66.4 | OVCAR-3 |
| 19.4 | 16.8 | 12.2 | OVCAR-4 |
| 30 | 16 | 40.3 | OVCAR-5 |
| 48.2 | 20.4 | 10.9 | OVCAR-8 |
| 71.6 | 24.3 | 3.3 | PC-3 |
| 14.5 | 17.1 | 6.85 | RPMI-8226 |
| 89 | 16.725 | 20.2 | RXF-393 |
| 40.7 | 25 | 12.1 | SF-268 |
| 60.8 | 22.4 | 8.12 | SF-295 |
| 49.4 | 22.3 | 4.92 | SF-539 |
| 28.8 | 32.65 | 66.6 | SK-MEL-2 |
| 16 | 32 | 322 | SK-MEL-28 |
| 19.5 | 67.1 | 19.5 | SK-MEL-5 |
| 22.9 | 15.35 | 14.1 | SK-OV-3 |
| 114 | 23.85 | 23.4 | SN12C |
| 21.4 | 19.65 | 11.3 | SNB-19 |
| 53.6 | 17.45 | 11 | SNB-75 |
| 23.2 | 18.32 | 4.23 | SR |
| 4.91 | 39.05 | 9.65 | SW-620 |
| 7.74 | 29.7 | 9.17 | T-47D |
| 86.7 | 19.805 | 135 | TK-10 |
| 113 | 19.9 | 12.1 | U251 |
| 25.8 | 27.3 | 86.7 | UACC-257 |
| 69.2 | 29 | 38.5 | UACC-62 |
| 41.4 | 16.535 | 104 | UO-31 |

**Supplementary Table 1. GNA13 mRNA expression positively correlates to mRNA expression of TIC/CSC marker ALDH1 in NCI-60 cancer cell line panel:** This data analysis is done using microarray data sets from NCI-60 panel cell lines. A Pearson correlation analysis was performed between the basal mRNA expression of GNA12 vs ALDH1 and GNA12 vs GNA13 and GNA13 vs ALDH1 and the p values are indicated in the table. The relative mRNA expression data is shown in the respective columns as indicated.
